# The effect of body contouring on the dose distribution delivered with volumetric‐modulated arc therapy technique

**DOI:** 10.1120/jacmp.v16i6.5810

**Published:** 2015-11-08

**Authors:** Jaegi Lee, Jong Min Park, Hong‐Gyun Wu, Jin Ho Kim, Sung‐Joon Ye

**Affiliations:** ^1^ Program in Biomedical Radiation Sciences Department of Transdisciplinary Studies, Seoul National University Graduate School of Convergence Science and Technology Seoul; ^2^ Biomedical Research Institute Seoul National University Hospital Seoul; ^3^ Department of Radiation Oncology Seoul National University Hospital Seoul; ^4^ Institute of Radiation Medicine Seoul National University Medical Research Center Seoul; ^5^ Center for Convergence Research on Robotics Advanced Institutes of Convergence Technology Suwon; ^6^ Department of Radiation Oncology Seoul National University College of Medicine Seoul Republic of Korea

**Keywords:** body contouring, volumetric‐modulated arc therapy, dose‐volumetric parameter, Hounsfield unit

## Abstract

The purpose of the study was to investigate the dosimetric effect defining the body structure with various Hounsfield unit (HU) threshold values on the dose distributions of volumetric‐modulated arc therapy (VMAT) plans. Twenty patients with prostate cancer and twenty patients with head and neck (H&N) cancer were retrospectively selected. For each patient, the body structure was redefined with HU threshold values of $-180({\text{Body}}_{180})$, $-350({\text{Body}}_{350})$, $-700({\text{Body}}_{700})$, and $-980({\text{Body}}_{980})$. For each patient, dose‐volumetric parameters with those body structures were calculated using identical VMAT plans. The differences in dose‐volumetric parameters due to the varied HU threshold values were calculated. For the prostate boost target volume, the maximum dose, mean dose, $D_{95\%}$, and $D_{5\%}$ with ${\text{Body}}_{180}$ were higher than those with ${\text{Body}}_{980}$ by approximately 0.7% (p<0.001). For H&N target volumes, the changes in $D_{95\%}$ of the targets receiving 67.5 Gy, 54 Gy, and 48 Gy between ${\text{Body}}_{180}$ and ${\text{Body}}_{980}$ were −1.2%, −0.9%, and −1.2%, respectively (p<0.001). The differences were larger for H&N VMAT plans than for prostate VMAT plans due to the inclusion of an immobilization device in the irradiated region in H&N cases. To apply all attenuating materials to dose calculation, the body structure would be defined with −980 HU. Otherwise, systematic error of about 1%, resulting in underdosage of the target volume, can occur.

PACS number: 87.55.ne

## I. INTRODUCTION

Since the introduction of intensity‐modulated radiation therapy (IMRT) in the field of radiation therapy, conformal delivery of a prescription dose to the target volume while minimizing dose to normal tissues has become possible.^(1)^ This has resulted in a reduction in radiation‐induced post‐therapy complications.^2^, ^3^, ^4^, ^5^ Volumetric‐modulated arc therapy (VMAT), as compared to IMRT, enables even faster and more efficient delivery of better or equivalent dose distributions.^(6)^ In VMAT, the gantry goes through single or multiple rotations while simultaneously modulating multileaf collimator (MLC) positions, dose rates, and gantry rotation speed.^6^, ^7^, ^8^, ^9^, ^10^

Both IMRT and VMAT are of benefit to the treatment of prostate cancer, as well as head and neck (H&N) cancer, since organs at risk (OARs) in both cases are located close to the target volume, which is irradiated by a typically high prescription dose.^4^, ^5^, ^11^, ^12^, ^13^ Many studies have demonstrated the clinical superiority of both IMRT and VMAT as compared to conventional non‐modulated modalities for the treatment of both prostate and H&N cancer.^2^, ^5^, ^11^, ^12^, ^13^ Furthermore, technological advances in dose calculation algorithm development have enabled more accurate evaluation of treatment plans.^14^, ^15^, ^16^, ^17^ Through utilization of state‐of‐the‐art techniques in radiation therapy, in terms of both calculation and delivery, highly conformal treatment of both prostate and H&N cancer has become possible.^2^, ^4^, ^5^, ^12^ However, elaborate treatments with steep dose gradients generated using IMRT and VMAT techniques are more susceptible to errors than are conventional radiation therapy treatments.^18^, ^19^ Since highly complex plans are routinely clinically performed for both H&N and prostate cancers, it is critical that every procedure be examined and evaluated carefully.^(20)^ These evaluation procedures are in place to detect major and minor factors which may hinder the accuracy of the delivered treatment.

In the treatment planning process, body structures must be defined in the treatment planning system (TPS) to facilitate generation of a plan. When calculating the dose distribution to be delivered to the patient, commercial TPS consider materials only inside the body structure, excepting supporting structures such as the treatment table.^(21)^ Therefore, in order to calculate dose distributions accurately, appropriate contouring of the body structure is essential in order to include all the materials affecting the treatment beam. When an immobilization device is used during treatment, it should also be applied to the patient during treatment simulation to acquire a CT image set for planning, and should also be included in the body structure to consider the extra attenuation caused by the immobilization device. In the cases of prostate or H&N VMAT, this should be considered even more carefully as OARs are close to the target volume, and therefore near the resulting steep dose gradients.^22^, ^23^ Furthermore, the photon beam attenuation and resulting alterations to the dose distribution caused by the immobilization device may be difficult to intuitively account for and challenging to predict, especially for VMAT. Regarding this, the American Association of Physicists in Medicine (AAPM) Task Group 176 (TG‐176) recently published a report which recommends contouring of the immobilization devices for consideration of their dosimetric effects.^(24)^ On the other hand, although the immobilization device is not applied, the body contour can be changed slightly according to the Hounsfield unit (HU) threshold values adopted to define the body structure in the TPS. In this case, changes in the volume of the body structure and the density of the increased body volume might be minimal. However, the dosimetric effects due to the change in the volume of the body structure remain unclear. Currently there exist no guidelines for how to define the body structure. This lack of a well‐defined set of guidelines could be potentially problematic for complex treatments such as prostate and H&N VMAT.

In this study, we investigated the effects of body contouring using various HU threshold values on clinically relevant dose‐volumetric parameters for both prostate and H&N VMAT plans. For each patient, the body structures defined with various HU threshold values were contoured, and the changes in dose‐volumetric parameters were investigated. The statistical significances of the differences in those dose‐volumetric parameters were analyzed.

## II. MATERIALS AND METHODS

### A. Patient selection and simulation

Twenty patients with prostate cancer and twenty patients with nasopharyngeal cancer (H&N cancer), who were treated using the VMAT technique in our institution, were retrospectively selected. The 40 total patients were chosen using a random selection process for this study. Each patient underwent CT simulation with Brilliance CT Big Bore (Philips, Cleveland, OH) in the supine position. The slice thickness of the CT images of patients with prostate cancer was 1.5 mm, and 3 mm for patients with H&N cancer. For the acquisition of CT images for prostate cancer, CT acquisition parameters of 120 kVp and 250 mAs/slice were used, while 120 kVp and 300 mAs/slice were used for H&N cancer. Patients with prostate cancer were immobilized with Smart Rest (Chunsung, Seoul, Republic of Korea), which is a combination of kneefix and feetfix, which allow the knee and feet positions of the patient to be repositioned. No immobilization device was applied in the region of irradiation for prostate cancer patients. Patients with H&N cancer were immobilized with a thermoplastic mask and a Silverman pillow (Bionix Radiation Therapy, Toledo, OH). In contrast to the immobilization devices for prostate cancer, the devices for H&N cancer were included in the region of irradiation during treatment.

### B. Treatment planning

All of the VMAT plans for patients with both prostate and H&N cancer were generated with the Eclipse system (Varian Medical Systems, Palo Alto, CA). For optimization of the VMAT plans, the progressive resolution optimizer 3 (PRO3, ver. 10, Varian Medical Systems) was used. For the calculation of dose distributions, the anisotropic analytic algorithm (AAA, ver. 10, Varian Medical Systems) was used. The dose calculation grid was 2 mm. TrueBeam STx with high‐definition MLC (Varian Medical Systems) was used for the prostate VMAT plans while Trilogy with Millennium 120 MLC (Varian Medical Systems) was used for the H&N VMAT plans. For prostate and H&N VMAT plans, 10 MV and 6 MV photon beams were used, respectively. For prostate plans, a primary plan with a primary target volume and a boost plan with a boost target volume were generated for each patient. The primary target volume was defined with a 2 cm margin in all directions from both the prostate and seminal vesicles except in the posterior and inferior directions. To the posterior and inferior directions, a 1 cm margin was added to reduce dose to the rectal wall. The boost target volume was defined with a 0.7 cm margin in all directions from the prostate only. A total of 50.4 Gy was delivered to the primary target volume with a daily dose of 1.8 Gy (28 fractions). After that, a total of 30.6 Gy was delivered to the boost target volume with a daily dose of 1.8 Gy (17 fractions). Rectal wall, bladder, and femoral heads were defined as OARs. Each of the prostate VMAT plans was generated with a single full arc. For the H&N VMAT plans, the simultaneous integrated boost (SIB) technique was used with a total of three target volumes. ${\text{Target}}_{67.5\text{Gy}}$, ${\text{target}}_{54\text{Gy}}$, and ${\text{target}}_{48\text{Gy}}$ were each generated with a margin of 0.3 cm in every direction. A total of 67.5 Gy with a daily dose of 2.25 Gy, 50.4 Gy with a daily dose of 1.8 Gy, and 48 Gy with a daily dose of 1.6 Gy were delivered to ${\text{target}}_{67.5\text{Gy}}$, ${\text{target}}_{54\text{Gy}}$, and ${\text{target}}_{48\text{Gy}}$, respectively (30 fractions). The brain stem, spinal cord, each eye, each lens, optic chiasm, and each parotid gland were defined as OARs and contoured. Each of the H&N VMAT plans was generated with two full arcs. For all patients in this study, four structure sets with four body structures were generated. The body structures were defined with HU threshold values of −180, −350, −700 and −980 (${\text{Body}}_{180},{\text{Body}}_{350},{\text{Body}}_{700}$, and ${\text{Body}}_{980}$, respectively). The value of −180 was chosen to include the light material of the human body since the HU of adipose with our CT simulator was −77, while the value of −980 was chosen to include the thermoplastic mask inside the body contour. The structures for all patients were identical, with the exception of the body structure. All holes present inside the body structure were filled. The VMAT plans for each patient were generated with ${\text{Body}}_{180}$ and dose distributions were calculated. The dose distributions with ${\text{Body}}_{350},{\text{Body}}_{700}$ and ${\text{Body}}_{980}$ were then calculated using identical VMAT plans as those generated with Body_180_. The original VMAT plans were simply generated with ${\text{Body}}_{180}$ since there is no guideline for defining body structure currently.

### C. Data analysis

Clinically significant dose‐volumetric parameters for each patient with ${\text{Body}}_{180},{\text{Body}}_{350},{\text{Body}}_{700}$, and ${\text{Body}}_{980}$ were calculated based on recommendations made in several previous studies.^25^, ^26^

For the target volume of prostate VMAT plans, parameters such as dose received by at least 95% volume of the boost target volume ($D_{95\%}$), $D_{5\%}$, the percent volume of boost target volume irradiated by at least 100% of the prescription dose ($V_{100\%}$), $V_{95\%}$, maximum and mean dose to the boost target volume, and the homogeneity index (*HI*) were calculated. The HI was calculated as follows:^(26)^

(1) $$Homogeneity\;index(HI)=\frac{D_{5\%}-D_{95\%}}{Mean\;dose\;to\;the\;\text{target}\;volume}$$

For the rectal wall, maximum and mean dose, $V_{47\text{Gy}}$, and $V_{75.6\text{Gy}}$ were calculated. For the bladder, mean dose, $D_{50\%}$, and $V_{47\text{Gy}}$ were calculated, while for femoral heads only the maximum dose was calculated.

For H&N VMAT plans, $D_{95\%},D_{5\%},V_{100\%},V_{95\%}$, maximum and mean dose for each target volume were calculated. The HI for ${\text{target}}_{67.5\text{Gy}}$ was also calculated. For OARs, the maximum dose to the brain stem, each lens, optic chiasm, and spinal cord, the mean dose to each parotid gland and each eye, and the values of $D_{50\%}$ of each parotid gland were calculated.

After calculation of dose‐volumetric parameters, the changes in those parameters according to HU threshold values of body structures were calculated as follows:

(2) $$Difference(\%)=\frac{DV_{n}-DV_{m}}{DV_{m}}\times 100$$

where $DV_{n}$ and $DV_{m}$ are a dose‐volumetric parameter with ${\text{Body}}_{n}$ and ${\text{Body}}_{m}$, respectively.

The statistical significances of the differences were analyzed using the paired *t*‐test.

### D. Measurements

The output of the 6 MV photon beams produced using Trilogy was calibrated with a measured value based on the AAPM TG‐51 protocol.^(27)^ After that, 500 monitor units (MU) were delivered with the newly calibrated Trilogy and the dose at the 10 cm depth was measured using a Farmer type ion chamber (Type 30010, PTW, Freiburg, Germany) inserted into a Solid Water phantom (Gammex, Middleton, WI). The source‐to‐surface distance (SSD) and field size were 100 cm and $10\times 10\;{\text{cm}}^{2}$, respectively. The measurements were each repeated 5 times. The CT image set of the Solid Water phantom was acquired and imported into the Eclipse system. The ${\text{Body}}_{180},{\text{Body}}_{350},{\text{Body}}_{700}$, and ${\text{Body}}_{980}$ of the Solid Water phantom were delineated, and 500 MU was delivered to those body structures in the TPS under the same conditions as the measurement. The calculated values of ${\text{Body}}_{180},{\text{Body}}_{350},{\text{Body}}_{700}$, and ${\text{Body}}_{980}$ were acquired and compared to the measured value.

## III. RESULTS

### A. Changes in the body structures due to HU threshold values

**Figure 1:**
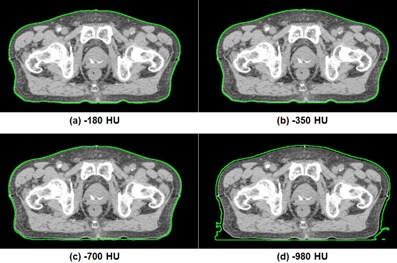
Body structures of a patient with prostate cancer defined with (a) −180 HU, (b) −350 HU, (c) −700 HU, and (d) −980 HU are shown, respectively. The body structures are shown in green line.

**Figure 2:**
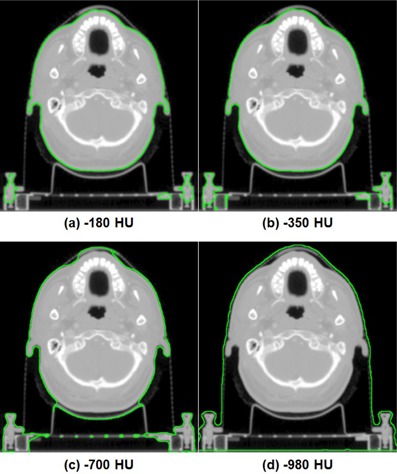
Body structures of a patient with head and neck cancer defined with (a) −180 HU, (b) −350 HU, (c) −700 HU, and (d) −980 HU are shown, respectively. The body structures are shown in green line.

Examples of changes in body structures according to HU threshold values of a patient with prostate cancer and a patient with H&N cancer are shown in Figs. 1 and 2, respectively. As the HU threshold values were increased from −980 to −180, the body structure of prostate and H&N cancer patients contracted by 13.8% and 48.8% on average, respectively. With increasing HU thresholds values, body contour contracted by 1−2 mm for prostate patients and 4−9 mm for H&N patients. The large change in the volume of body structure of H&N patients with decreasing HU thresholds values was due to inclusion of immobilization devices. Especially, the oral cavity area showed large differences between body threshold values of −980 and −180. Of the body structures for patients with H&N cancer, only ${\text{Body}}_{980}$ fully included the immobilization devices.

### B. Changes in dose‐volumetric parameters of VMAT plans for prostate cancer

**Figure 3:**
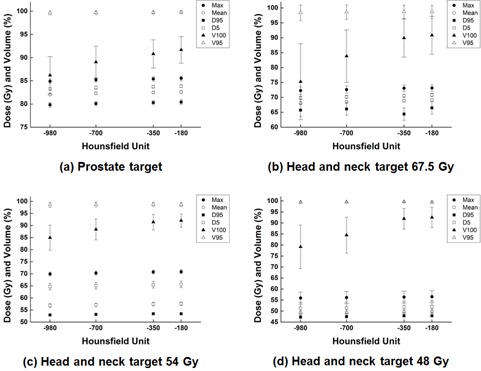
The changes for prostate cancer (a) in the maximum and mean dose to the target volume, dose delivered to the 95% of target volume ($D_{95\%}$) and $D_{5\%}$, the percent volume of the target volume received 100% of prescription dose ($V_{100\%}$), and $V_{95\%}$ of volumetric‐modulated arc therapy (VMAT) plans. Same parameters as prostate cancer are shown for head and neck cancer of target 67.5 Gy (b), target 54 Gy (c), and target 48 Gy (d).

The dose‐volumetric parameters of the prostate boost target volume calculated with ${\text{Body}}_{180},{\text{Body}}_{350},{\text{Body}}_{700}$, and ${\text{Body}}_{980}$ are shown in Fig. 3. The differences between the body structure and the associated p‐values are shown in Table 1. Although most of the differences were not large, showing differences less than 1%, all differences with the exception of HI were statistically significant with all p‐values less than 0.002. The maximum dose, mean dose, $D_{95\%}$, and $D_{5\%}$ with ${\text{Body}}_{180}$ were higher than those with ${\text{Body}}_{980}$ by approximately 0.7% (p<0.001). In the case of $V_{100\%}$, significant differences between ${\text{Body}}_{180}$ and ${\text{Body}}_{980}$ were observed (−6.0% with p<0.001), while the changes in $V_{95\%}$ according to the HU threshold values were not large (−0.15% with p<0.001). For prostate OARs, the differences in dose‐volumetric parameters between the body structures are shown in Table 2. Similar results to those of the target volume were observed for OARs generally. In the case of $V_{75.6\text{Gy}}$ of the rectal wall, the difference between ${\text{Body}}_{180}$ and ${\text{Body}}_{980}$ was up to 6%, although the change in volume was only 0.4%. Because the values of $V_{75.6\text{Gy}}$ were relatively small (7.2% at ${\text{Body}}_{180}$), the percent differences were exaggerated. As the HU threshold values used to define the body structure were increased, dose‐volumetric parameters generally increased slightly. Although the magnitudes of the differences were not generally large, they were generally statistically significant.

**Table 1:** The differences in dose‐volumetric parameters of prostate boost target volume calculated with body contour defined by various HU thresholds values

	−180 vs. −350		−180 vs. −700		−180 vs. −980		−350 vs. −700		−350 vs. −980		−700 vs. −980	
	*Diff. (%)*	*p*	*Diff. (%)*	*p*	*Diff. (%)*	*p*	*Diff. (%)*	*p*	*Diff. (%)*	*p*	*Diff. (%)*	*p*
Maximum dose	−0.14±0.10	<0.001	−0.40±0.13	<0.001	−0.75±0.14	<0.001	−0.26±0.10	<0.001	−0.60±0.13	<0.001	−0.34±0.10	<0.001
Mean dose	−0.14±0.08	<0.001	−0.39±0.08	<0.001	−0.70±0.07	<0.001	−0.25±0.02	<0.001	−0.56±0.05	<0.001	−0.31±0.04	<0.001
$D_{95\%}$	−0.15±0.08	<0.001	−0.40±0.08	<0.001	−0.72±0.08	<0.001	−0.25±0.02	<0.001	−0.58±0.05	<0.001	−0.32±0.05	<0.001
$D_{5\%}$	−0.14±0.08	<0.001	−0.39±0.08	<0.001	−0.70±0.08	<0.001	−0.25±0.02	<0.001	−0.56±0.05	<0.001	−0.31±0.05	<0.001
$V_{100\%}$	−0.96±0.66	<0.001	−2.91±1.22	<0.001	−6.02±2.19	<0.001	−1.96±0.73	<0.001	−5.11±1.82	<0.001 1	−3.21±1.21	<0.001
$V_{95\%}$	−0.03±0.03	<0.001	−0.07±0.07	<0.001	−0.15±0.14	<0.001	−0.04±0.04	0.002	−0.12±0.12	<0.001	−0.08±0.08	<0.001
HI	0.18±0.43	0.091	0.28±0.41	0.013	0.54±0.92	0.036	0.11±0.48	0.437	0.36±0.82	0.120	0.26±0.86	0.259

HU=Hounsfield unit; Diff.=difference; $D_{n\%}=\text{dose received by the n}\%\text{volume of structure}$; $V_{n\%}=\text{percent volume irradiated at least n}\%\text{of prescription dose}$; HI=homogeneity index defined as (D5%−D95%)/mean dose.

**Table 2:** The differences in dose‐volumetric parameters of prostate organs at risk calculated with body contour defined by various HU thresholds values

	−180 vs. −350		−180 vs. −700		−180 vs. −980		−350 vs. −700		−350 vs. −980		−700 vs. −980	
	*Diff. (%)*	*p*	*Diff. (%)*	*p*	*Diff. (%)*	*p*	*Diff. (%)*	*p*	*Diff. (%)*	*p*	*Diff. (%)*	*p*
*Rectal Wall*												
Maximum dose	−0.15±0.18	0.002	−0.42±0.26	<0.001	−0.72±0.14	<0.001	−0.26±0.15	<0.001	−0.57±0.10	<0.001	−0.31±0.18	<0.001
Mean dose	−0.11±0.07	<0.001	−0.31±0.08	<0.001	−0.52±0.08	<0.001	−0.20±0.05	<0.001	−0.41±0.07	<0.001	−0.21±0.04	<0.001
$V_{47\text{Gy}}$	−0.22±0.15	<0.001	−0.61±0.21	<0.001	−1.01±0.26	<0.001	−0.39±0.13	<0.001	−0.79±0.16	<0.001	−0.41±0.10	<0.001
$V_{75.6\text{Gy}}$	−1.29±1.00	<0.001	−3.62±1.66	<0.001	−6.15±2.37	<0.001	−2.37±0.91	<0.001	−4.93±1.66	<0.001	−2.63±1.08	<0.001
*Bladder*												
Mean dose	−0.09±0.08	0.001	−0.28±0.08	<0.001	−0.49±0.09	<0.001	−0.19±0.03	<0.001	−0.40±0.06	<0.001	−0.21±0.04	<0.001
$D_{50\%}$	0.06±0.25	0.835	0.03±0.30	0.104	0.04±0.39	0.056	−0.04±0.12	0.014	−0.02±0.29	0.026	0.01±0.18	0.048
$V_{47\text{Gy}}$	−0.18±0.12	0.056	−0.54±0.22	<0.001	−0.94±0.37	<0.001	−0.36±0.14	<0.001	−0.76±0.30	<0.001	−0.41±0.17	0.001
*Femoral Head*												
Maximum dose	−0.15±0.30	0.073	−0.45±0.41	<0.001	−0.78±0.36	<0.001	−0.30±0.27	<0.001	−0.63±0.20	<0.001	−0.33±0.26	<0.001

HU=Hounsfield unit; Diff.=difference; $D_{n\%}=\text{dose received by the n}\%\text{volume of structure}$; $V_{\text{nGy}}=\text{percent volume irradiated at least n Gy}$.

### C. Changes in dose‐volumetric parameters of VMAT plans for H&N cancer

The dose‐volumetric parameters of H&N target volumes calculated with ${\text{Body}}_{180},{\text{Body}}_{350},{\text{Body}}_{700}$, and ${\text{Body}}_{980}$ are shown in Fig. 3. For the target volumes, the differences of the body structures along with associated p‐values are shown in Table 3. The changes in $D_{95\%}$ of ${\text{target}}_{67.5\text{Gy}}$, ${\text{target}}_{54\text{Gy}}$, and ${\text{target}}_{48\text{Gy}}$ between ${\text{Body}}_{180}$ and ${\text{Body}}_{980}$ were −1.2%, −0.9%, and −1.2%, respectively (all with p<0.001). In the case of $V_{100\%}$, significant differences between ${\text{Body}}_{180}$ and ${\text{Body}}_{980}$ were observed (−17.4% for ${\text{target}}_{67.5\text{Gy}}$, −7.7% for ${\text{target}}_{54\text{Gy}}$, and −14.4% for ${\text{target}}_{48\text{Gy}}$) with statistical significances (p<0.001), while the changes in $V_{95\%}$ according to the HU threshold values were not large with no statistical significances (p>0.05). For H&N OARs, the differences in dose‐volumetric parameters between the body structures and the associated p‐values are shown in Table 4. The differences between ${\text{Body}}_{180}$ and ${\text{Body}}_{980}$ in the maximum dose to the brain stem, spinal cord, left and right lens, and optic chiasm were −1.8%, −1.5%, −3%, −3.4%, and −1.2%, respectively (all with p>0.03). The differences between ${\text{Body}}_{180}$ and ${\text{Body}}_{980}$ in the mean doses to left and right eyes were −1.9% and −1.5% (p>0.022 and 0.002, respectively). The magnitudes of the differences in H&N VMAT plans were larger than those of prostate VMAT plans, due to the presence of the immobilization devices in the irradiated region of H&N VMAT plans.

**Table 3:** The differences in dose‐volumetric parameters of head and neck target volumes calculated with body contour defined by various HU thresholds values

	−180 vs. −350		−180 vs. −700		−180 vs. −980		−350 vs. −700		−350 vs. −980		−700 vs. −980	
	*Diff. (%)*	*p*	*Diff. (%)*	*p*	*Diff. (%)*	*p*	*Diff. (%)*	*p*	*Diff. (%)*	*p*	*Diff. (%)*	*p*
${\text{Target}}_{67.5\text{Gy}}$												
Maximum dose	−0.09±0.68	0.555	−0.68±0.81	0.002	−1.17±0.97	<0.001	−0.59±0.41	<0.001	−1.08±0.56	<0.001	−0.50±0.32	<0.001
Mean dose	−0.29±0.24	<0.001	−0.87±0.27	<0.001	−1.45±0.30	<0.001	−0.58±0.30	<0.001	−1.16±0.27	<0.001	−0.58±0.26	<0.001
$D_{95\%}$	−0.11±0.28	0.316	−0.69±0.41	<0.001	−1.24±0.45	<0.001	−0.59±0.17	0.419	−1.13±0.27	0.514	−0.55±0.22	<0.001
$D_{5\%}$	−0.28±0.21	<0.001	−0.87±0.39	<0.001	−1.36±0.53	<0.001	−0.59±0.22	<0.001	−1.09±0.39	<0.001	−0.50±0.26	<0.001
$V_{100\%}$	−0.82±1.56	0.030	−7.70±4.75	<0.001	−17.43±10.30	<0.001	−6.96±4.21	<0.001	−16.79±10.11	<0.001	−10.87±7.79	<0.001
$V_{95\%}$	0.06±0.24	0.263	−0.05±0.42	0.629	−0.27±0.59	0.069	−0.10±0.24	0.049	−0.32±0.46	0.006	−0.22±0.24	0.002
HI	−2.61±3.50	0.006	−2.70±4.94	0.020	−1.62±6.52	0.198	−0.10±3.10	0.828	1.02±5.40	0.412	1.06±2.20	0.038
${\text{Target}}_{54\text{Gy}}$												
Maximum dose	−0.13±0.48	0.247	−0.75±0.60	<0.001	−1.29±0.63	<0.001	−0.62±0.35	<0.001	−1.16±0.45	<0.001	−0.55±0.31	<0.001
Mean dose	−0.22±0.11	<0.001	−0.80±0.14	<0.001	−1.26±0.18	<0.001	−0.58±0.08	<0.001	−1.04±0.14	<0.001	−0.46±0.10	<0.001
$D_{95\%}$	−0.01±0.19	0.811	−0.47±0.35	<0.001	−0.93±0.35	<0.001	−0.46±0.18	<0.001	−0.92±0.20	<0.001	−0.46±0.10	<0.001
$D_{5\%}$	−0.23±0.30	0.004	−0.90±0.34	<0.001	−1.44±0.42	<0.001	−0.67±0.13	<0.001	−1.21±0.27	<0.001	−0.56±0.22	<0.001
$V_{100\%}$	−0.63±1.20	0.033	−3.96±2.84	<0.001	−7.70±3.98	<0.001	−3.38±1.80	<0.001	−7.14±3.12	<0.001	−3.92±1.62	<0.001
$V_{95\%}$	0.14±0.21	0.016	0.05±0.36	0.727	−0.15±0.49	0.205	−0.09±0.18	0.038	−0.29±0.33	0.002	−0.20±0.16	<0.001
${\text{target}}_{48\text{Gy}}$												
Maximum dose	−0.29±0.32	0.001	−0.74±0.92	0.002	−1.09±1.00	<0.001	−0.45±0.64	0.006	−0.81±0.77	<0.001	−0.35±0.79	0.069
Mean dose	−0.26±0.50	0.031	−1.07±0.52	<0.001	−1.47±0.60	<0.001	−0.80±0.18	<0.001	−1.21±0.28	<0.001	−0.41±0.17	<0.001
$D_{95\%}$	−0.04±0.43	0.666	−0.82±0.52	<0.001	−1.23±0.58	<0.001	−0.78±0.27	<0.001	−1.19±0.33	<0.001	−0.41±0.13	<0.001
$D_{5\%}$	−0.35±0.23	<0.001	−1.02±0.43	<0.001	−1.32±0.56	<0.001	−0.68±0.27	<0.001	−0.98±0.40	<0.001	−0.31±0.20	<0.001
$V_{100\%}$	−0.41±4.74	0.596	−8.60±7.50	<0.001	−14.36±9.70	<0.001	−8.31±4.67	<0.001	−14.15±7.23	<0.001	−6.50±3.87	<0.001
$V_{95\%}$	0.17±0.22	0.002	0.08±0.31	0.245	0.00±0.35	0.949	−0.08±0.11	0.006	−0.17±0.17	0.001	−0.09±0.06	<0.001

HU=Hounsfield unit; Diff.=difference; $D_{n\%}=\text{dose received by the n}\%\text{volume of structure}$; $V_{n\%}=\text{percent volume irradiated at least n}\%\text{of prescription dose}$; HI=homogeneity index defined as (D5%−D95%)/mean dose.

**Table 4:** The differences in dose‐volumetric parameters of head and neck organs at risk calculated with body contour defined by various HU thresholds values

	−180 vs. −350		−180 vs. −700		−180 vs. −980		−350 vs. −700		−350 vs. −980		−700 vs. −980	
	*Diff. (%)*	*p*	*Diff. (%)*	*p*	*Diff. (%)*	*p*	*Diff. (%)*	*p*	*Diff. (%)*	*p*	*Diff. (%)*	*p*
Max. to BS	−0.32±0.36	0.001	−1.09±0.34	<0.001	−1.79±0.39	<0.001	−0.77±0.23	<0.001	−1.47±0.31	<0.001	−0.71±0.37	<0.001
Mean. to eye (L)	−1.10±3.00	0.148	−1.60±3.01	0.045	−1.87±3.02	0.022	−0.50±0.21	<0.001	−0.78±0.32	<0.001	−0.28±0.13	<0.001
Mean. to eye (R)	−0.75±1.78	0.083	−1.21±1.81	0.009	−1.47±1.86	0.002	−0.47±0.20	<0.001	−0.73±0.27	<0.001	−0.26±0.13	<0.001
Max. to lens (L)	−1.34±3.03	0.123	−2.63±3.22	0.003	−3.01±3.46	0.002	−1.31±0.71	<0.001	−1.69±1.04	<0.001	−0.39±0.67	<0.001
Max. to lens (R)	−1.81±3.43	0.061	−2.91±3.60	0.006	−3.39±3.73	0.002	−1.12±0.71	<0.001	−1.62±1.04	<0.001	−0.51±0.52	<0.001
Max. to OC	−0.40±0.76	0.017	−0.79±0.87	<0.001	−1.23±1.10	<0.001	−0.38±0.41	<0.001	−0.83±0.65	<0.001	−0.45±0.50	<0.001
Mean. to PG (L)	−0.51±2.96	0.424	−0.49±3.02	0.439	−0.60±3.05	0.380	0.02±0.20	0.789	−0.09±0.38	0.305	−0.11±0.26	0.062
Mean. to PG (R)	−0.54±3.05	0.467	−0.52±3.07	0.491	−0.63±3.09	0.399	0.02±0.23	0.693	−0.09±0.45	0.434	−0.11±0.28	0.119
$D_{50\%}$ to PG (L)	−0.15±2.35	0.654	0.21±2.48	0.971	0.33±2.57	0.817	0.35±0.34	<0.001	0.47±0.59	0.002	0.12±0.49	0.134
$D_{50\%}$ to PG (R)	−0.04±1.83	0.942	0.36±1.92	0.293	0.53±2.01	0.174	0.40±0.32	<0.001	0.57±0.61	0.001	0.17±0.45	0.120
Max. to SC	−0.43±0.72	0.018	−0.97±0.73	<0.001	−1.48±0.70	<0.001	−0.54±0.20	<0.001	−1.05±0.26	<0.001	−0.51±0.26	<0.001

HU=Hounsfield unit; Diff.=difference; Max.=maximum dose; BS=brain stem; Mean.=mean dose; (L)=left; (R)=right; OC=optic chiasm; PG=parotid gland; $D_{n\%}=\text{dose received by the n}\%\text{volume of structure}$; SC=spinal cord.

### D. Measurements vs. calculations with ${\text{Body}}_{180}$ and ${\text{Body}}_{980}$

The measured dose with the ion chamber in a Solid Water phantom was 328.7±0.04 cGy. The calculated dose acquired with ${\text{Body}}_{180},{\text{Body}}_{350},{\text{Body}}_{700}$, and ${\text{Body}}_{980}$ were 330.1 cGy, 329.3 cGy, 328.8 cGy, and 328.6 cGy, respectively. The differences between the measurement and calculations for ${\text{Body}}_{180},{\text{Body}}_{350},{\text{Body}}_{700}$, and ${\text{Body}}_{980}$ were 0.43%, 0.18%, 0.03%, and −0.03%, respectively.

## IV. DISCUSSION

In this study, the dosimetric effect due to the contouring of the body structure was investigated for prostate and H&N VMAT plans. As the HU threshold values used to define the body structure were increased, nearly all dose‐volumetric parameters increased due to a decrease in the attenuating material. As shown in Figs. 1 and 2, the volume of the body increased when HU threshold values decreased, which meant an increase in attenuating material. Additionally, immobilization devices with low density, such as thermoplastic masks, can be fully included in the body structure with −980 HU threshold value. In order to follow the recently published AAPM TG‐176 protocol, when contouring the body structure, the HU threshold value should be lower than −980 to include the immobilization device (i.e., thermoplastic mask, pillow, or vacuum bag) in the body structure.^(24)^ The measured value was more coincident with the calculated values with ${\text{Body}}_{700}$ and ${\text{Body}}_{980}$ than were the others. It seems appropriate to use the body structure defined with −980 HU.

When adopting HU threshold values of −980 to define the body structure, the blurred boundaries of CT images were also included in the body structure, showing a different SSD from the real SSD in the TPS. When adopting −980 as a HU threshold value for body contouring, this should be kept in mind.

In the case of $V_{100\%}$ of both prostate and H&N VMAT, a considerable discrepancy was observed between ${\text{Body}}_{180}$ and ${\text{Body}}_{980}$ of up to 17%. However, since the values of $V_{95\%}$ were not changed significantly, the large change in values of $V_{100\%}$ seems to not be clinically significant. The large change in $V_{100\%}$ and small change in $V_{95\%}$ mean that the dose gradients were steeper around the isodose line of 100% of prescribed dose than were those around the 95% isodose, resulting in sensitive changes in $V_{100\%}$ due to small changes in dose.

If we use HU threshold values higher than −980, such as −180, to define the body structure, the calculated dose in the TPS for evaluation of treatment plan quality would show higher values than the dose which would actually be delivered to the patient. Therefore, as the delivered dose would be lower than calculated, the dose to the OARs would not be problematic. However, the coverage of the target volume by the prescription dose in the calculation would be shown better than actual delivery. The delivered prescription dose would be 0.7% less than expected without an immobilization device, or 1% with an immobilization device such as a thermoplastic mask. Although the changes in dose‐volumetric parameters were small, which could potentially be clinically insignificant, we believe it is valuable to eliminate errors in the course of radiotherapy for more accurate treatment of the patient, regardless of the magnitudes of the errors. Moreover, the dosimetric error demonstrated in this study was a systematic error which could widely influence the quality of radiation therapy.

## V. CONCLUSIONS

For the definition of body structures in TPS for treatment planning, an appropriate HU threshold value should be used in order to consider all material including immobilization devices with low density during dose calculation. In this study, HU threshold value of −980 seems appropriate for the definition of body structures for both prostate and H&N VMAT plans.
